# Lab protocol for investigating impacts of climate change on frogs

**DOI:** 10.1016/j.mex.2022.101767

**Published:** 2022-06-20

**Authors:** Muhammad Saeed, Muhammad Rais, Syeda Maria Ali, Durraj Nadeem Khosa, Ayesha Akram, Waseem Ahmed, Sumbul Gill

**Affiliations:** a Herpetology Lab, Department of Wildlife Management, Pir Mehr Ali Shah Arid Agriculture University Rawalpindi, Punjab, Pakistan, 46000; b Assistant Professor, Environmental Sciences, International Islamic University Islamabad, Islamabad, 44000; c Fatima Jinnah Women University Rawalpindi, Rawalpindi, Pakistan, 46000

**Keywords:** Dicroglossidae, Climate change, Extinction, Threatened, Data deficient, Survival

## Abstract

We developed a method to investigate impacts of temperature (elevated) on breeding, growth and development in endemic frogs under laboratory conditions. The method provides details on housing and rearing of larvae, taking various important measurements and observing developmental deformities. The method could also be applied to rescue approach or head-start program for amphibian species experiencing climate change elsewhere in the world.

• Rearing of larvae to investigate effects of temperature on larvae

• Measurement of data on growth and development

• Rescue/ head-start program

## Graphical abstract

**Figure:**
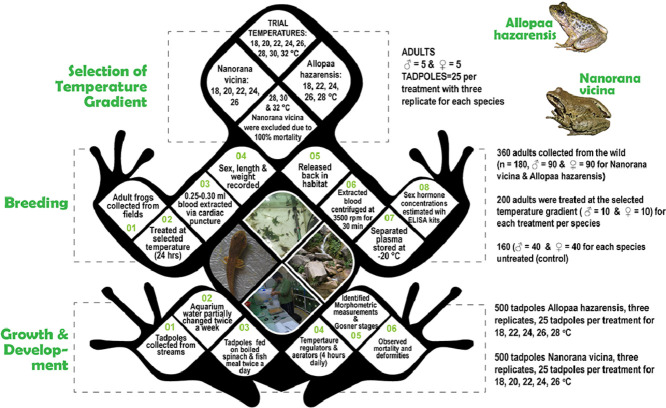

Specifications table

**Table:** 

Subject Area:	Agricultural and Biological Sciences
More specific subject area:	Wildlife Biology
Method name:	Lab protocol for investigating impacts of climate change on frogs
Name and reference of original method:	*N.A*
Resource availability:	*N.A*

## Method details

Collect the larvae and adult frogs (males and females) from the wild and run trial experiments for the selection of temperature gradient. Exclude the temperatures that cause 100% mortality.

**Fig. 1:**
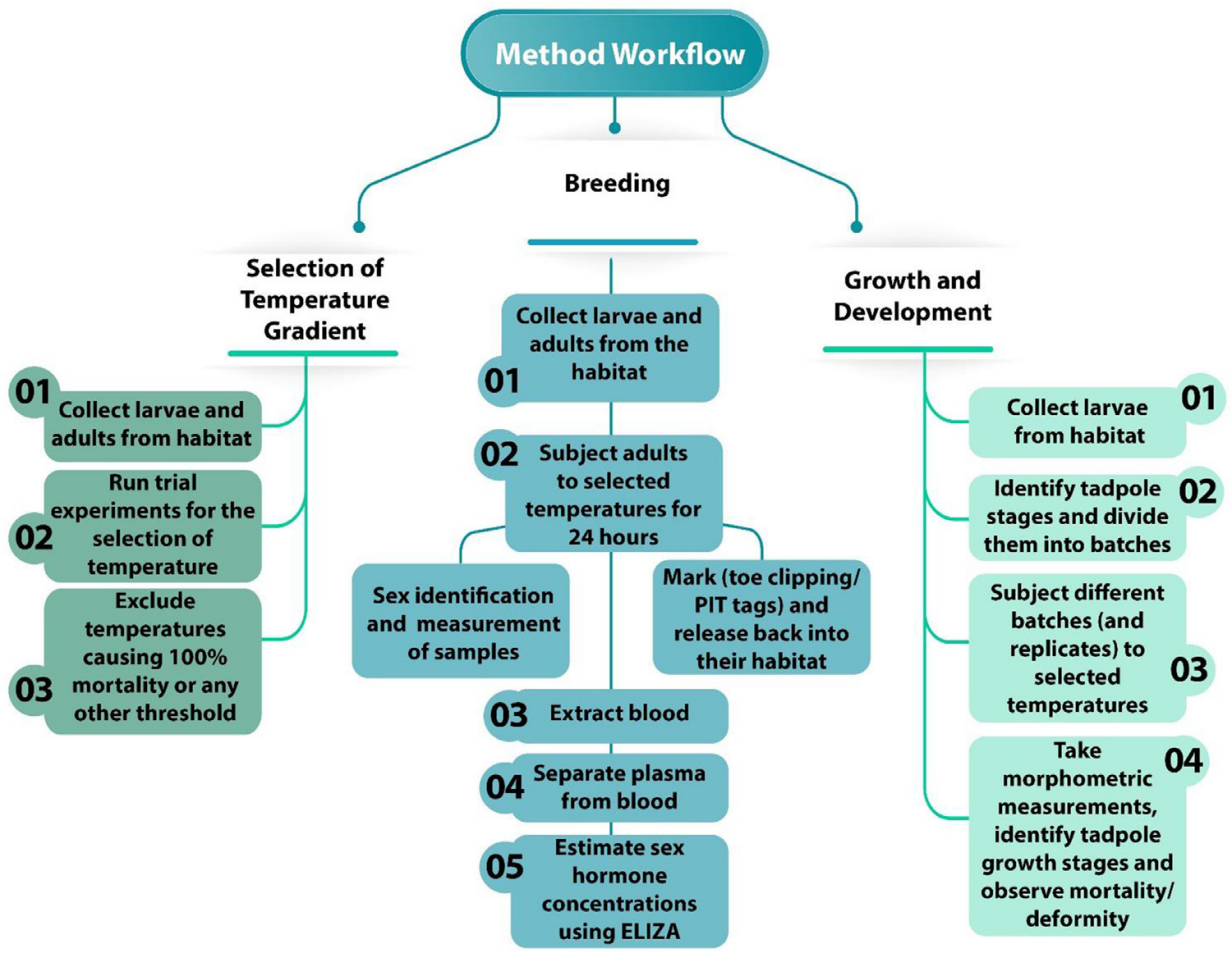
Flow chart showing main steps involved in the method developed to examine impacts of climate change on frogs.

For the estimation of sex hormones (**♀** estradiol, **♂** testosterone), keep them in plastic bucket for 24 hours. Collect the blood through cardiac puncture using an insulin syringe (30 gauge, 1 mL/cc) and store it in EDTA (K3EDTA) tubes. Separate the plasma by centrifuging the blood for 30 minutes at 3000 rotations per minute. Determine the sex of the individual by examining secondary sexual characters. Measure the snout-vent length and weight using a digital vernier caliper and a weighing balance, respectively. Mark the frog (toe-clip/PIT tag or any other method) and release them back to their habitats. Perform the enzyme linked immunosorbent assay (ELISA) for the estimation of sex hormones by following the kit protocol (Fig. 1).

Capture the larvae from habitats using dip net and choose larvae of a desired Gosner stage for the experiments by subjecting different batches (and replicates) to the selected temperatures. Rear the larvae in glass aquaria filled with tap water. Control the aquaria temperature using temperature regulators, an aerator with a stone for air and a filter (kept on for four hours/day). Feed larvae twice a day with boiled spinach/vegetable and supplement with fishmeal. Record the development stages (till 46) once a week and observe percentage mortality/ survival on daily basis. Take the morphometric measurement [total length, snout vent length (SVL), tail length, body width (max.), tail length (max.), body height, tail height (max.),total height (max.) and body mass] of tadpoles once a week. Examine the larvae for gross morphological deformities such as edema (swelling of abdomen) and tail kink (twist/curve in tail) or any other.

## Method validation

We successfully applied this method to study if elevated temperatures had any effect on breeding, growth development and fitness of Hazara Torrent Frog (*Allopaa hazarensis*) and Murree Hills Frog (*Nanorana vicina*) (Saeed et al., 2021) [1]. We performed trials for the selection of temperature gradient. We subjected five adult males and females and 25 tadpoles (Gosner stage 25) of *A. hazarensis* and *N. vicina* for 24 h to temperatures 18, 20, 22, 24, 26, 28, 30 and 32°C. We excluded temperatures 30 and 32 °C for *A. hazarensis* and 28, 30 and 32 °C for *N. vicina* due to 100% mortality at these temperatures.

We collected 180 adult frogs of the two studied species (♂ = 90, ♀ = 90) from freshwater streams and springs of the study area (Murree, District Rawalpindi, Province Punjab and Ayubia National Park, Khyber Pakhtunkhwa) from February 2018 to October 2019. We subjected 200 adults to the selected temperatures (♂ = 10, ♀ = 10 for each temperature/ species) and kept 160 (♂ = 40, ♀ = 40/ species) as control. We pooled the data for the months of March-April (spring, temperature of water = 14 °C), May-June (summer, 23 °C), July-August (monsoon, 22 °C), and September-October (fall, 11 °C).

We exposed the frogs for 24 hours at selected temperatures in plastic buckets (L x W x H, 45 × 35 × 48 cm) at field station near the frog habitat. We extracted the blood through cardiac puncture using insulin syringe (30 gauge, 1 mL/cc) and stored it in EDTA (K3EDTA) tubes. We sexed each frog by inspecting the belly and thumb. Females have soft belly and no nuptial pad on the thumb. We used digital caliper (INSIZE Electronic Calipers series 1112 (0.01 mm) and weighing balance 0.01 g (g) to measure snout-vent length and weigh the frogs, respectively. We then marked by toe clipping and released frogs back into their habitats. Finally, we extracted the plasma by centrifuging blood for 30 mintues at 3000 rotations per minute (rpm). We performed enzyme linked immunosorbent assay (ELISA) by following the kit protocol Fish Estradiol (E2) ELISA kit, Catalogue No. SL0033FI; Fish Testosterone ELISA kit, Catalogue No. SL0032FI).

We collected larvae (Gosner stage 25) of *A. hazarensis* and *N. vicina* using dip nets. We divided the larvae into five batches (100 larvae per batch, 25 larvae per replication) for each species and reared them in the glass aquaria (L × W × H, 38 × 30 × 42 cm) filled with 15 L tap water. The water temperature was maintained by using temperature regulator (16–34 °C ± 0.5), an aerator with a stone for air circulation and a filter (kept on for four hours/day). The larvae were fed twice in a day with boiled spinach and supplemented with fish meal. We recorded development stages (GS 25–46) once a week. The duration of trials was 21 and 24 weeks for the larvae of *A. hazarensis* and *N. vicina*, respectively. To determine mortality, number of dead tadpoles was counted on daily basis. We recorded the morphometric measurements [total length, snout vent length (SVL), tail length, body width (max.), tail length (max.), body height, tail height (max.),total height (max.) and body mass] of tadpoles once a week [1]. We followed Wolf et al. (1969) [2] and Chinathamby et al. [3] to examine gross morphological deformities such as edema (swelling of abdomen) and tail kink (twist/curve in tail).

## Declaration of Competing Interest

The authors declare that they have no known competing financial interests or personal relationships that could have appeared to influence the work reported in this paper.

## Data Availability

All data were deposited in NCBI Genbank. Accession numbers are provided in the main text.

## Appendix Supplementary materials

Image, application 1
