# Supplementary material for: Lab protocol for investigating impacts of climate change on frogs
Source: MethodsX. 2022 Jun 20;9:101767. doi: 10.1016/j.mex.2022.101767 (PMC9256650; doi:10.1016/j.mex.2022.101767)
Supplement: Supplementary file 1 [file mmc1.pdf]

Pir Mehr Ali Shah
ARID AGRICULTURE UNIVERSITY RAWALPINDI
Institutional Ethics Committee (IEC)

No. PMAS-AAUR/IEC/ 281

Dated: 18-12-2017

CERTIFICATE OF APPROVAL

Title of Protocol:	Breeding, growth and population monitoring of endemic anurans under changing climate in Murree and Ayubia National Park, Pakistan
Principal Investigator:	Muhammad Saeed
Department:	Wildlife Management, Pir Mehr Ali Shah Arid Agriculture University Rawalpindi, Murree Road, Rawalpindi

The proposed research was reviewed in the meeting held on 15th December, 2017. The model species are of Least Concern population trend according to IUCN (International Union for Conservation of Nature and Natural Resources) and are currently "Not Protected" under any local wildlife law. The committee is, therefore, pleased to grant approval for the execution of this study for the period of **02 (two) years**.

It was agreed that animal ethics guideline publishes as Second Edition, revised by the Herpetological Animal Care and Use Committee (HACC) of the American Society of Ichthyologists and Herpetologists, 2004. (Committee Chair: Steven J. Beaupre, Members: Elliott R. Jacobson, Harvey B. Lillywhite, and Kelly Zamudio) will be adopted and adhere to strictly.

Prof. Dr. Mazhar Qayyum
Convener, IEC
Dean Faculty of Sciences

Institutional Ethics Committee (IEC), PMAS-Arid Agriculture University Rawalpindi, Pakistan.
Phone : +92-51-9290467, E-mail : drmazhar.qayyum@uaar.edu.pk

Scanned with CamScanner
